# Supplementary material for: Prevalence of, and risk factors for, diabetes and prediabetes in Bangladesh: Evidence from the national survey using a multilevel Poisson regression model with a robust variance
Source: PLOS Glob Public Health. 2022 Jun 1;2(6):e0000461. doi: 10.1371/journal.pgph.0000461 (PMC10021925; doi:10.1371/journal.pgph.0000461)
Supplement: Attachment — Submitted filename: Response to Editor and Reviwers.docx [file pgph.0000461.s002.docx]

**Reviewers’ comments and author’s responses**

| **Reviewers’ comments** | **Authors’ response** |
| --- | --- |
| **Reviewer #1** |  |
| This study aims to assess the prevalence and associated factors of diabetes and prediabetes among Bangladeshi adults. This study will provide some evidence for literature review. However, some shortage should be addressed: | Thank you. We have updated the manuscript addressing your comments and suggestions. A point-by-point response is provided below. |
| Discussion on the condition of diabetes among several divisions should have been added as you have mentioned "administrative division" as an associated factor. | We have added a paragraph in the discussion section to address this issue. This reads*-*  *The likelihood of diabetes was high among respondents of the Dhaka division and low among respondents of the Rangpur and Barishal divisions. Many factors might contribute to such differences in risks of diabetes across divisions. For instance, being the capital of Bangladesh, the Dhaka division is highly urbanised and inhabited by people with high levels of education. Consequently, sedentary lifestyles and dependency on western foods are highly prevalent among residents of Dhaka as compared to the residents of other divisions in Bangladesh. Dhaka is also commonly characterised by very poor environmental conditions, including extreme air pollution, and few parks and open spaces. Together these factors contribute to an increased risk of diabetes among residents of Dhaka division. In contrast, the Rangpur division is mostly rural, and people are largely employed in agricultural activity. These characteristics could be responsible for a lower risk of diabetes among residents of the Rangpur division. These regional variations in diabetes and prediabetes prevalence in Bangladesh are consistent with studies conducted in other South Asian countries, including India and Nepal [43, 44].* |
| Some explanation should be added on the selection of references to determine the risk factors of diabetes and prediabetes. | We have added an explanation about confounding variable selection in the ‘Explanatory variables’ section of the methods. This now reads-  *Three different levels of explanatory variables of diabetes and pre-diabetes were identified through a comprehensive review of the literature [5, 9, 10, 17].* |
| **Reviewer#2** |  |
| Thank you for the opportunity to review this manuscript. This paper addresses an important topic about the assessment of prevalence of, and risk factors for, diabetes and prediabetes in Bangladesh using the national survey. However, there are several critical insufficiencies in this manuscript have to be addressed. |  |
| Minor comments |  |
| 1. Authors skipped a crucial risk factor of diabetes, cigarette smoking which should be justified. In short, lifestyle behaviour (alcohol consumption, smoking status, sleep duration, diet, and physical activity level etc.) should be acknowledged. If not, then the reason must be justified. | We agree with the reviewer that these are crucial risk factors for diabetes and should be considered in the model. However, relevant data were not collected in the survey. We have acknowledged this limitation both in the methods and study limitation section. We have added the following sentences in the ‘Explanatory variables’ section of the Methods section-  *The literature review also identified family history of diabetes, mass media exposure, cigarette smoking, alcohol consumption, sleep duration, diet, and physical activity as significant behavioral and lifestyle-related individual-level risk factors of diabetes and pre-diabetes. However, this study could not include these variables in the analyses as they were not included in the original survey.*  We have added the following sentences in the strength and limitation section-  *Behavioral factors (e.g., alcohol consumption, smoking status, sleep duration), dietary factors (e.g., type and amount of food taken), physical activity, and genetic factors (e.g., family history of diabetes) are crucial risk factors for diabetes. However, these data were not collected and could not be controlled for in the analyses.* |
| 2. Also, family history is an established risk factor of diabetes which has been clearly avoided in this paper. | Please see our response above. |
| 3. Mass media exposure can influence the lifestyle and behavioral pattern of an individual which in turn has direct impact on diabetes. This paper didn’t address mass media exposure as an associated factor. | We agree with the reviewer that mass media exposure is an important predictor of lifestyle and behavioral patterns. However, in the Bangladesh Demographic and Health Survey (BDHS), mass media exposure data were collected only from women aged 15-49 years. Mass media exposure data for women aged above 49 years, and male respondents, were not available in the BDHS. We, therefore, did not include this variable in our analyses.  Additionally, mass media messages generally target a particular health outcome. For example, in the BDHS, data has been collected for exposure to family planning messages through the mass media (radio, television, newspaper). However, no data is collected following mass media messages targeting diabetes in the BDHS. This is a limitation of the BDHS dataset. |
| 4. In page 9, line 258-259, it is mentioned that “individuals aged ≥50 years were about three times more likely to have diabetes (Table 4)”. 60-64 age group has the similar result. Authors need to focus on this issue and put more emphasis and describe in the discussion section. | In this revised version of the manuscript, we have drawn attention to these findings and added relevant explanations and citations. The paragraph in the discussion now reads-  *There is a well-established relationship between increasing age and risk of diabetes which is consistent with our study [4, 27]. The risks are even higher among the respondengts’ aged ≥50 years. The most important factors leading to such increasing risks are (i) deficiency of insulin secretion developing with age and (ii) growing insulin resistance caused by a change in body composition and sarcopaenia [28, 29]. The implication is that with increasing life expectancy in Bangladesh (current life expectancy at birth 72.3 years) [30], the increasing numbers of older people will result in even more cases and a higher burden of diabetes.* |
| Major comments |  |
| Materials and Method (Page 4, Line 124-125)  In this study, it’s not clear if you have 2 separate models for diabetes and prediabetes or not. In this case, it is no problem to categorize study subjects into diabetes and non-diabetes, but if you categorize the same subjects into prediabetes and non-prediabetes it’s not appropriate. If so, then it will combine diabetes and non-prediabetes into one single group which doesn’t make sense for your study objective. | We have developed two final adjusted models. We dichotomized ‘diabetes’ into people with diabetes or no diabetes (including prediabetes). We dichotomized ‘prediabetes’ into people with prediabetes or no diabetes. Therefore, we excluded respondents with diabetes (either from numerator or denominator) while the variable ‘prediabetes’ was constructed. We have now edited the relevant sentences in the Methods to make this clear. The new sentences now read-  *The World Health Organization (WHO) criteria for diabetes and prediabetes classification were used [16]. The variable diabetes included respondents with diabetes defined as FPG level greater than or equal to 7.0 mmol/L or those reporting using medication for diabetes. Respondents without diabetes were those with FPG levels less than 7.0 mmol/L and not taking any diabetes controlling medication. The variable prediabetes included respondents with prediabetes defined as FPG levels between 6.1 mmol/L and 6.9 mmol/L and not taking diabetes controlling medication. Respondents without prediabetes were those with FPG levels less than 6.1 mmol/L and not taking diabetes controlling medication [16].*  Additionally, we have added a sub-section entitled “Analytic sample” under the Methods section, where we have clarified the sample analysed for both diabetes and prediabetes. It reads-  *Of the respondents who had their blood glucose tested, we excluded those for whom body mass index data were missing (n=143) and those who were pregnant at the time of blood glucose measurement (n=7). After exclusion, we had 11,952 respondents who had their blood glucose tested which was our analytical sample for analysing prevalence and risk factors of diabetes. However, in the subsequent analyses of -pre-diabetes prevalence and risk factors, respondents having diabetes (n=1,174) were excluded, and the data for the remaining 10,779 respondents were analysed.* |
| 2. It is suggested to show the trend of diabetes and prediabetes in Bangladesh to make this manuscript more informative | National level data on diabetes and pre-diabetes are only available in the 2011 and 2017/18 Bangladesh Demographic and Health Survey. Showing trends using only two time points data is very unusual. We, therefore, cannot address this comment. However, in the Introduction and Discussion sections, we have reported the increasing trend of diabetes and prediabetes in Bangladesh based on systematic reviews that included national and regional level cross-sectional data. For instance, in the Introduction section, we have the following sentences-  *Studies, including a systematic review and meta-analysis, and national survey reports, showed that the prevalence of diabetes among adults had increased substantially in Bangladesh, from ~5% in 2001 to ~14% in 2017(5-8). However, these studies are relatively old, and overlooked the prevalence of, and risk factors for, prediabetes*  In the Discussion section, we have the following sentences-  *In 2021, the IDF ranked Bangladesh 8^th^ of countries with the highest number of adults (20–79 years) with diabetes (13.1 million cases), and it is expected to be ranked 7^th^ in 2045 (4), consistent with our estimates. In our analysis, about 1 in 10 adults (18+) had diabetes, representing an estimated over 7.9 million individuals in Bangladesh in 2020.* |
| **Overall Comment**  *Authors need to justify the necessity of this particular study when there exist similar studies as it does not appear to add anything new to what is already known about diabetes and prediabetes in Bangladesh (ref: Sarker AR 2021).* | Thank- you for identifying the study by Sarker and Khanam 2021. We note that these authors submitted their manuscript on 26 August 2021, 3 weeks after we submitted our manuscript on 4 August 2021. This means we could not have referred to their study in our paper. However, we have now reviewed the article by Sarker and Khanam (2021) and have identified the following:   - Sarker and Khanam (2021) included respondents with gestational diabetes and/or pre-diabetes in their estimation of diabetes prevalence. We do not believe this is correct and may have led to overestimating the burden of diabetes disease in Bangladesh. The BDHS excluded this sample from their analyses and the survey report. Therefore, Sarker and Khanam’s (2021) paper sample size is different from the sample considered in the respective survey report. - Sarker and Khanam (2021) included respondents with missing body mass index data. - Sarker and Khanam (2021) did not use any standardization (i.e., age) in estimating the burden of diabetes and pre-diabetes. However, we have presented age-standarized estimates for diabetes and pre-diabetes. Thus, we believe our assessment of the burden of diabetes and prediabetes is more robust than Sarker and Khanam (2021), as our analysis takes into account the age structure of the population. - Sarker and Khanam (2021) estimated the odds ratio for diabetes using logistic regression [13, 14]. We used Poisson regression with robust variance in our manuscript that corrects the overestimation of risks for diabetes and pre-diabetes. Poisson regression is more robust when the outcome is common, and we believe it is the correct analytic approach for the current data. - Sarker and Khanam (2021) have used the same variables twice in their logistic regression model, which is highly problematic. For example, they have used ‘possession of Television and Radio’ in constructing the Wealth Index. Then, they again used these two variables to measure mass media exposure. - Finally, Sarker and Khanam (2021) have included two lifestyle-related variables of limited value in determining epidemiological exposure as covariates. Specifically, they have used the following two variables in Table 1-4: (a) Smoked/used tobacco and (b) Caffeinated drink. The original questions used to collect these data are: “Have you done any of the following within the past 30 minutes: Had coffee, tea, cola or other drink that has caffeine; Smoked/used tobacco?” Questions that captured long-term exposure to these factors would have been more beneficial in determining causality.   For these reasons, we think our manuscript adds new information to Sarker and Khanam 2021 about diabetes and prediabetes in Bangladesh. Importantly, our study addresses several of the design flaws in the Sarker and Khanam 2021 study and, as such, does make an original and important contribution to the literature. |
