# Supplementary material for: Prevalence of, and risk factors for, diabetes and prediabetes in Bangladesh: Evidence from the national survey using a multilevel Poisson regression model with a robust variance
Source: PLOS Glob Public Health. 2022 Jun 1;2(6):e0000461. doi: 10.1371/journal.pgph.0000461 (PMC10021925; doi:10.1371/journal.pgph.0000461)
Supplement: Attachment — Submitted filename: Response to Editor and Reviwers.docx [file pgph.0000461.s003.docx]

**Reviewers’ comments and author’s responses**

| **Reviewers’ comments** | **Authors’ response** |
| --- | --- |
| **Reviewer #1** |  |
| Line 127; "and the data for the" should be replaced by "and the data of the". | All comments have been addressed in the specified lines. |
| Line 143; "or those reporting" should be replaced by "or those reported". |  |
| Line 147; "any" should be added after "and not taking". |  |
| Line 149; "any" should be added after "not taking". |  |
| Line 357; "respondengts" spelling should be corrected. |  |
| Line 401; "divisions" should be changed to "division". |  |
| Line 404; "lifestyles" should be changed to "lifestyle". |  |
